# Effects of Black Soldier Fly Larvae Oil on Growth Performance, Blood Biochemical Parameters, Carcass Quality, and Metabolomics Profile of Breast Muscle of Thai Native Chickens

**DOI:** 10.3390/ani14213098

**Published:** 2024-10-27

**Authors:** Theeraphat Srikha, Padsakorn Pootthachaya, Warin Puangsap, Nisakon Pintaphrom, Nantanant Somparn, Wuttigrai Boonkum, Anusorn Cherdthong, Bundit Tengjaroenkul, Sawitree Wongtangtintharn

**Affiliations:** 1 Department of Animal Science, Faculty of Agriculture, Khon Kaen University, Khon Kaen 40002, Thailand; theeraphat.sr@kkumail.com (T.S.); padsakornp@kkumail.com (P.P.); nisakon_m@kkumail.com (N.P.); nantanant.s@kkumail.com (N.S.); wuttbo@kku.ac.th (W.B.); anusornc@kku.ac.th (A.C.); 2 Department of Veterinary Public Health, Faculty of Veterinary Medicine, Khon Kaen University, Khon Kaen 40002, Thailand; warin.paun@kkumail.com (W.P.); btengjar@kku.ac.th (B.T.); 3 Network Center for Animal Breeding and Omics Research, Khon Kaen University, Khon Kaen 40002, Thailand

**Keywords:** black soldier fly larvae, insect oil, performance, metabolomics profile, Thai native chicken

## Abstract

This study aimed to evaluate the effects of the replacement of rice bran oil (RBO) with black soldier fly larvae oil (BSFLO) on growth performance, blood biochemicals, carcass quality, and metabolomics profile of breast muscle of Thai native chickens. A total of 192 1-day-old, mixed-sex, Pradu Hang Dam (Mor Kor 55) chickens were randomly allocated to one of three dietary groups. Each treatment had four replicates with 16 chicks per replicate (8 males and 8 females). Three dietary treatments were used: (T1) the control group, based on a corn–soybean meal with RBO, and two treatment groups that replaced 50% (T2) and 75% (T3) of RBO in the basal diet with BSFLO, respectively. Results showed that BSFLO inclusion at 50% and 75% did not adversely affect the productive performance of Thai native chickens (*p* > 0.05). Regarding blood profiles, on day 28, chickens fed 75% BSFLO exhibited significant increases in hemoglobin, hematocrit, and MCHC (mean corpuscular hemoglobin concentration) with lower eosinophil percentages compared to the control group (*p* < 0.05). Additionally, BSFLO supplementation raised glucose levels but decreased globulin and total protein levels (*p* < 0.05). On day 63, BSFLO inclusion primarily affected MCV (mean corpuscular volume), with higher values in the 50% BSFLO group (*p* < 0.05). It also increased globulin and HDL (high-density lipoprotein) levels while lowering AST (aspartate transaminase) concentrations (*p* < 0.05). For carcass and meat quality, BSFLO supplementation did not affect dressing percentage or edible meat yield (*p* > 0.05). However, it influenced meat pH and shear force (*p* < 0.05), with a higher pH at 24 h post-mortem in BSFLO-fed chickens. Metabolomics showed that arginine biosynthesis; phenylalanine, tyrosine, and tryptophan metabolism; alanine, aspartate, and glutamate metabolism; arginine and proline metabolism; and taurine and hypotaurine metabolism were the most differentially abundant. These findings suggest that BSFLO can be used for a partial replacement (50 to 75%) for RBO in Thai native chicken diets, potentially offering benefits for animal health and meat quality without compromising growth performance.

## Simple Summary

This research was conducted to evaluate the suitability of black soldier fly larvae oil (BSFLO) as a feedstuff for Thai native chickens (Pradu Hang Dam Mor Kor 55). The BSFLO, a sustainable and nutrient-rich alternative, offers several benefits, including improved meat quality, enhanced blood parameters, and positive effects on muscle metabolism. Although BSFLO did not have any significant effect on the growth of the chickens, it demonstrated potential for improving the overall health and value of indigenous chicken products. Further research is required to explore the long-term economic viability and scalability of BSFLO as a feed ingredient for Thai native chickens.

## 1. Introduction

The growing demand for sustainable and efficient poultry production has prompted researchers to explore alternative feed ingredients that can enhance the health and productivity of native chicken breeds. Thai native chickens have the potential to produce meat with functional health benefits, offering improved nutritional and bioactive properties. Several years prior, enhancements in the performance of Thai native chickens regarding growth and egg production were realized by the Research and Development Network Center for Animal Breeding (Native Chicken), Khon Kaen University, Thailand [1]. Pradu Hang Dam Mor Kor 55 showed exceptional growth performance and was highly valued for their hardiness, resistance to diseases, and adaptability to local conditions [1,2]. Additionally, the native chicken contains nutrients and bioactive compounds beneficial to human health, including L-carnitine, creatine, carnosine, and anserine [1]. Despite these advantages, these chickens often exhibit slower growth rates and lower productivity compared to commercial broilers, making it challenging to meet the increasing market demand. As a result, there is a pressing need to identify feed additives or feed ingredients that can improve the growth performance and overall quality of native chicken breeds while maintaining their inherent advantages.

One such promising feed ingredient is black soldier fly larvae (BSFL) oil, which is rich in essential nutrients and has become a topic of great interest in recent years for its high nutritional content and sustainability. Black soldier fly larvae oil (BSFLO) mainly consists of medium-chain saturated fatty acids, with lauric acid being the dominant component. Numerous studies reported that the lipid composition of black soldier fly larvae is influenced by the composition of their growth media [3,4], but lipid content will always be dominated by lauric acid. Several other studies also revealed that BSFLO has a high lauric acid content and is of similar quality to coconut oil and palm kernel oil.

Several other studies have also revealed that BSFLO has a lot of advantages over plant-origin oil, such as a viable dietary energy source that can replace oil commonly used in livestock feed. Furthermore, it is well documented that BSF larvae fat not only provides energy but may also be considered a functional feed material [5,6]. In particular, the high concentration of lauric acid as a predominant medium-chain fatty acid (MCFA) in BSF helps abdominal fat reduction because of its preferential use in energy utilization over long-chain saturated or unsaturated fatty acids. In addition, it may positively affect the microbiota microecosystem of the birds’ gastrointestinal tracts [7,8]; additionally, an indirect effect on immune system support [5] and metabolic efficiency [9] can be observed. Furthermore, the production of black soldier fly larvae is environmentally friendly, as these insects can efficiently convert organic waste into valuable biomass, making BSFLO a cost-effective and eco-friendly alternative to traditional feed ingredients [10].

Former studies demonstrated the potential of BSFLO in improving the growth performance and health of various livestock species [9,11,12]. Another study investigated the effects of BSFLO on broiler chickens and found that it might be a promising substitute for dietary fat [13]. However, its effects on native chicken breeds, particularly the Pradu Hang Dam Mor Kor 55, remain underexplored. Understanding how BSFLO influences key aspects such as growth performance, blood biochemical parameters, carcass quality, and the metabolomics profile is crucial for optimizing its use in poultry production. These investigations would provide a platform for the discovery of the possible advantages of BSFLO for the development of the productivity and quality of Thai native chickens.

This study aimed to evaluate the effects of black soldier fly larvae oil on the growth performance, blood biochemical parameters, carcass quality, and metabolomics profile of breast muscle of Thai native chickens. By examining these aspects, we sought to determine whether BSFLO can serve as a viable alternative feed ingredient that not only supports sustainable poultry production but also adds value to the native chicken industry in Thailand. The findings of this research could have significant implications for the development of more efficient and sustainable poultry feeding strategies, contributing to the overall improvement of native chicken breeds.

## 2. Materials and Methods

### 2.1. Animal Ethics

All of the experimental protocols and procedures used in this research were reviewed and approved by the Institutional Animal Care and Use Committee of Khon Kaen University (record no. IACUC-KKU-61/66), based on the Ethics of Animal Experimentation of the National Research Council of Thailand.

### 2.2. Sample Preparation

Black soldier fly larvae oil (BSFLO) in this study was purchased from BSFLY Company Ltd., located in Udon Thani, Thailand. The BSFLO was extracted using an NF-80 cold press oil machine (Karaerler in Ankara, Turkey); detailed information regarding the BSF oil extraction process was described in the previous work of Prachumchai and Cherdthong [14]. Then, the energy content of the BSFLO was analyzed using an adiabatic bomb calorimeter (AC500, LECO Corp., Michigan, IN, USA); it was 4047.60 kcal/kg. This energy value was utilized to assess the effects of BSFLO in dietary feed in animal trials within the current study.

### 2.3. Animals and Experimental Design

A total of 192 Pradu Hang Dam (Mor Kor 55) chickens, an indigenous strain developed by the Network Center for Animal Breeding and Omics Research, Faculty of Agriculture, Khon Kaen University, Thailand [1], were used in this study. The 1-day-old, mixed-sex chicks (male-to-female ratio 1:1) were randomly distributed into one of three treatment groups. Each dietary treatment had four replicate pens, with each pen consisting of 16 birds (8 males and 8 females), following a completely randomized design. Three dietary treatments were used: (T1) the control group, based on a corn–soybean meal with rice bran oil (RBO), and two treatment groups that replaced 50% (T2) and 75% (T3) of the RBO in the basal diet with BSFLO, respectively. The constituents and chemical composition of the diets (either analyzed or calculated values) in the different feeding phases are shown in Table 1. The diets were formulated to meet the nutritional needs of native chickens and followed a three-phase feeding program throughout the 63-day trial, including phase 1 (1–28 days), phase 2 (29–42 days), and phase 3 (43–63 days). The chickens were provided with pelleted feed and had unlimited access to the feed and water throughout the experiment. Rice hull was used as the bedding material. All birds were raised in pens with a total area of 4.20 m^2^. The chickens were exposed to 18 h of light and 6 h of darkness daily.

### 2.4. Data Collections

#### 2.4.1. Growth Performance and Economic Return

The performance of the birds was monitored throughout the study. Body weights were recorded individually for each bird in each pen on days 1, 28, 42, and 63, and body weight gain (BWG) was subsequently calculated. Feed intake was also recorded, and the feed conversion ratio (FCR) was calculated and adjusted for mortality. The number of dead birds was recorded to calculate the mortality rate. Economic returns were evaluated by calculating the following metrics: feed cost (FC), feed cost per gain (FCG), salable bird return (SBR), net profit return (NPR), and return on investment (ROI).

#### 2.4.2. Blood Profiles

On days 28 and 63, 12 birds (one bird per replicate) were randomly selected for blood collecting (5 mL) after a 12 h fasting period. Of this, 1 mL of each blood sample was placed in an EDTA-containing vial for hematological analysis; i.e., red blood cell (RBC), white blood cell (WBC), hematocrit, hemoglobin, mean corpuscular volume (MCV), mean corpuscular hemoglobin (MCH), and mean corpuscular hemoglobin concentration (MCHC) were determined using an automated hematology analyzer (Sysmex XE-2100, Kobe, Japan).

A separate 4 mL blood sample was collected in an EDTA-free vial and centrifuged to obtain serum. Individual serum samples were then analyzed for glucose, albumin, globulin, total protein, triglyceride, cholesterol, high-density lipoprotein (HDL), low-density lipoprotein (LDL) cholesterol, and hepatic enzymes, aspartate transaminase (AST) and alkaline phosphatase (ALP), which were measured by an auto-chemistry analyzer (Chiron, Emeryville, CA, USA).

#### 2.4.3. Carcass and Meat Quality

At the end of the feeding trial, after a 12 h fasting period, 48 birds (four birds per replicate) were euthanized. Pre-slaughter weights were recorded for each bird. The entire hot carcasses were weighed, then immediately eviscerated and sectioned into breast fillet, breast fillet inner, whole wing, thigh, and drumstick. Visceral and edible meat organs were weighed to calculate carcass yield. Within 10 min post-mortem, breast muscles were divided into two portions: one part (left side) was harvested and stored at 4 °C for subsequent meat quality analysis and another (right side) was immediately frozen at −20 °C for later metabolomics analysis.

The pH, surface color, drip loss, cooking loss, and texture profile analysis (TPA) were examined on meat samples from the left side of the breast meat, in accordance with the methodology defined by Malila et al. [15], with minor modifications. Briefly, breast meat pH was measured at 24 h post-mortem by directly inserting a spear-shaped probe equipped with a pH meter (Ion 510, Eutech Instruments Pte Ltd., Singapore) into three designated positions (cranial, medial, and caudal) of each raw meat sample. Subsequently, surface color was measured using a handheld colorimeter (CR-410 Series, Konica Minolta Sensing Inc., Tokyo, Japan). CIE L*, a*, and b* color parameters were determined by averaging three readings taken from three distinct spots on the breast meat surface.

Thereafter, the meat was weighed, enveloped in many layers of gauze cloth, and suspended at 4 °C for 24 h. The meat was gently patted dry using a paper towel and subsequently reweighed. The weight variation prior to hanging (W1) and subsequent to hanging (W2) was utilized to quantify drip loss (Equation (1)) as a percentage. The meat was subsequently cooked through water immersion at 80 °C until the core temperature of the deepest part of the breast reached 72 °C. The cooked meat was subsequently chilled in running tap water and allowed to equilibrate at 4 °C for 2 h prior to reweighing. The weight difference between the weights before (W1) and after (W2) cooking was used to express culinary loss (Equation (1)). The weight of the meat before drip loss determination and after cooking was different, as indicated by the total processing loss (%).

Drip loss or cooking loss (%) = [W1 − W2]/[W1] × 100(1)

The cooked meat was prepared for two different texture analyses: texture profile analysis (TPA) and Warner–Bratzler Shear (WBS) force test. For TPA, the meat was cut into 1 cm × 1 cm × 1 cm cubes and double-compressed, while for the WBS test, it was cut into 1 cm × 2 cm × 1 cm specimens and sheared perpendicularly to muscle fiber alignment. Both analyses were performed using a texture analyzer (model TA.HDplusC, Stable Micro Systems, Goldalming, UK) equipped with either a 50 mm cylindrical aluminum probe for TPA or a V-slot WB Blade for the WBS test. The Exponent software version 6.2.1.0 (Stable Micro Systems, Goldalming, UK) automatically calculated and reported all textural parameters for both analyses.

#### 2.4.4. Breast Metabolomics

Breast meat samples were processed for metabolite analysis using an untargeted LC-MS-based metabolomics approach. Each 0.1 g sample was extracted and mixed with methanol (0.1:1.0, *w*/*v*) in a microcentrifuge tube and then homogenized, sonicated, and centrifuged at 14,100 g for 5 min. The supernatant was transferred and filtered through a 0.22 µm membrane to prepare the sample for LC-MS analysis, with 100 µL of the extracted sample added to LC vials. Quality control (QC) samples were prepared by mixing 20 μL aliquots from each sample to monitor analytical deviations from these pool mixtures, while a blank was prepared using 100 µL of methanol [16].

Metabolomic profiles were obtained using an ACQUITY UPLC^®^ system combined with UPLC-based hydrophilic interaction liquid chromatography (HILIC) and a hybrid quadrupole time-of-flight (Q-Tof™) mass spectrometer (Agilent Technologies, Santa Clara, CA, USA), utilizing a BEH amide column (2.1 × 150 mm, 1.7 μm; Waters Corporation, Milford, MA, USA). Samples were injected and separated by HILIC at a flow rate of 0.4 mL/min at 45 °C, using two mobile phases: A (acetonitrile + 0.1% formic acid) and B (water + 0.1% formic acid). The gradient elution was programmed as follows: 99% A-1% B (0–0.1 min), 30% A-70% B (7.0 min), 99% A-1% B (7.1–10.0 min), with a total run time of 10 min. The QC samples were injected every 3 samples to assess repeatability. Mass spectrometry was performed using electrospray ionization in both positive and negative ion modes. Untargeted metabolite screening and identification were performed using Kyoto Encyclopedia of Genes and Genomes (KEGG) databases (http://www.genome.jp/kegg/, accessed on 22 July 2024).

### 2.5. Statistical Analysis

Data analysis was conducted using a one-way ANOVA within the general linear model (GLM) procedure of SAS [17]. All parameters were evaluated using a completely randomized design. Duncan’s multiple range test was applied to determine significant differences between treatment means, with statistical significance set at *p* < 0.05.

The MS-DIAL software (version 5.3.240328) was used to perform principal component analysis (PCA). Metabolites with a variable importance in projection (VIP) greater than 1 and a *p*-value less than 0.05 were considered significantly different. These differentially expressed metabolites were categorized and mapped into their corresponding biochemical pathways.

## 3. Results

### 3.1. Performance

The dietary replacement of RBO with BSFLO at both 50% and 75% did not influence (*p* > 0.05) the productive performance of Thai native chickens (Pradu Hang Dam Mor Kor 55) across all measured parameters and periods (Table 2).

The economic return of Thai native chickens (Pradu Hang Dam Mor Kor 55) throughout 63 days was not affected by the dietary treatment *(p* > 0.05; Table 3).

### 3.2. Blood Profiles

The blood and serum parameters of Thai native chickens are shown in Table 4 and Table 5. Results revealed significant improvements in several hematological and biochemical markers. On day 28, hemoglobin, hematocrit, and MCHC levels increased significantly in chickens fed 75% BSFLO compared to the control group (*p* < 0.05). Additionally, eosinophil percentage was significantly lower in the 75% BSFLO-fed group (*p* < 0.05). Regarding blood biochemical parameters, BSFLO supplementation led to a significant increase in glucose levels (*p* < 0.05), while globulin and total protein levels were significantly lower than the non-BSFLO-fed group (*p* < 0.05).

On day 63, the blood hematology of native chickens was not affected by dietary treatment, except for the MCV with a higher concentration in the 50% BSFLO group (*p* < 0.05). Regarding blood biochemical parameters, BSFLO inclusion significantly increased globulin and HDL levels (*p* < 0.05). Also, the AST concentrations were significantly lower in the BSFLO-fed group (*p* < 0.05).

### 3.3. Carcass and Meat Quality

For carcass quality, the dressing percentage and edible meat were unaffected by the dietary treatment (*p* > 0.05). For some of the internal organs, the percentage of the liver was highest in the control group (*p* < 0.05), while the percentage of the heart was significantly higher than the non-BSFLO-fed group (*p* < 0.05; Table 6).

The pH of the meat at 24 h post-mortem was significantly higher in chickens fed BSFLO (*p* < 0.05) compared to the control group. In terms of color, lightness (L*) and redness (a*) were not significantly affected, while yellowness (b*) was significantly lower (*p* < 0.05) in the BSFLO groups. Notably, the shear force was significantly influenced by BSFLO supplementation (*p* < 0.05), with the 50% BSFLO group showing the lowest value *(*Table 7).

### 3.4. Breast Metabolomics

**Figure 1:**
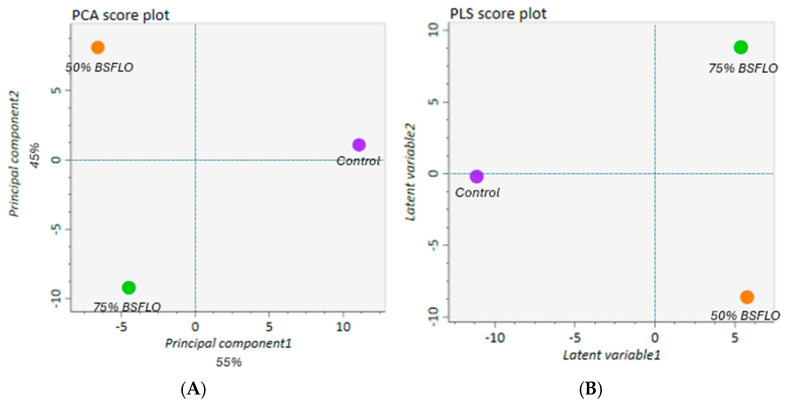
Metabolomics profile analysis of Thai native chicken (Pradu Hang Dam Mor Kor 55). (**A**) PCA (principal component analysis). (**B**) PLS-DA (partial least squares discrimination analysis). Groups are abbreviated as: Control (basal diet without BSFLO); BSFLO 50% and BSFLO 75% (basal diet with 50% and 75% replacement of RBO by BSFLO, respectively).

**Figure 2:**
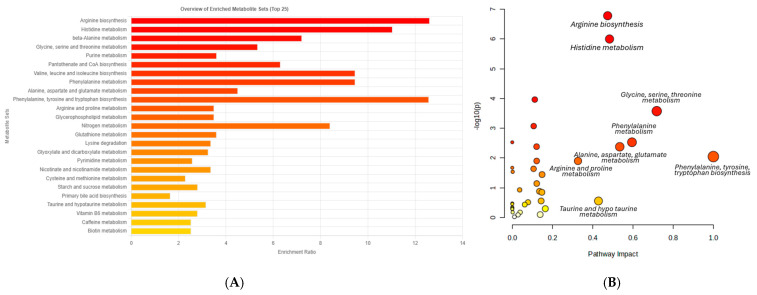
Pathway classification and topology analysis of Thai native chicken (Pradu Hang Dam Mor Kor 55). (**A**) Pathway classification of all significant metabolites of chickens fed control diet and 50 and 75% BSFLO diet. (**B**) KEGG topology analysis of the identified metabolites. Groups are abbreviated as: Control (basal diet without BSFLO); BSFLO 50% and BSFLO 75% (basal diet with 50% and 75% replacement of RBO by BSFLO, respectively).

Using UPLC-MS, 14,351 peaks were identified in the breast muscle samples of the control and BSFLO groups. Principal component analysis (PCA) and partial least squares-discriminant analysis (PLS-DA) revealed a distinct clustering of the control, 50% BSFLO, and 75% BSFLO groups in the score plots (Figure 1A,B). The metabolic profiles of the muscle tissues in the control and BSFLO groups were significantly different. Out of the 5140 metabolites that varied among the three groups, 176 were identified. Of these identified metabolites, 78 were up-regulated and 98 were down-regulated in the BSFLO groups. All significant metabolites were categorized into 22 Kyoto Encyclopedia of Genes and Genomes (KEGG) pathways. Among these pathways, amino acid metabolism was identified as the top priority (Figure 2A). KEGG topology analysis revealed that five metabolic pathways had a pathway impact value greater than 0.1, indicating their significance (Figure 2B). These metabolic pathways include (1) arginine biosynthesis; (2) phenylalanine, tyrosine, and tryptophan metabolism; (3) alanine, aspartate, and glutamate metabolism; (4) arginine and proline metabolism; and (5) taurine and hypotaurine metabolism. The metabolites within these metabolic pathways are listed in Table 8. Five differentially expressed metabolites were integrated into the KEGG pathway analysis.

## 4. Discussion

In the present study, the partial replacement of RBO with 50 and 75% of BSFLO for Thai native chickens had no detrimental effect on growth performance. The inclusion of 75% BSFLO also did not negatively affect growth performance compared to 50% BSFLO inclusion. This result shows the possibility of total replacement of RBO by BSFLO. Previous research also reported that including BSFLO in a broiler diet did not affect growth performance [7,8]. Through these results, it is possible to replace more than 75% of the RBO with the BSFLO in the native chicken diet in terms of growth performance.

In this study, we examined the economic implications of replacing RBO with BSFLO in the diet of Thai native chickens at 50% and 75% inclusion levels. Despite the nutritional benefits offered by BSFLO and its lack of adverse effects on growth performance, cost is a crucial factor that may influence its commercial viability. The results indicated that the cost of BSFLO is currently higher than that of RBO, especially at higher inclusion rates. However, the potential benefits of BSFLO, such as improved meat quality and reduced environmental impact, may offset the additional feed costs [18]. Further research is needed to evaluate the long-term economic viability of incorporating BSFLO into the diets of Thai native chickens, particularly as production costs and market demand for BSFLO-enriched products evolve. Despite these challenges, the use of the component of BSFL as an animal feed ingredient has significant potential to contribute to a more sustainable and resilient food system in Thailand, which has been actively promoting the use of BSFL as a sustainable animal feed ingredient. The government recognizes the potential benefits of BSFL, including its ability to reduce food waste, improve animal health, and contribute to a more circular economy [18,19].

The observed increase in hematocrit, MCHC, and MCV likely reflects improved red blood cell production and quality. This may be attributed to the higher medium-chain fatty acid content of BSFLO compared to RBO. The MCFA (medium-chain fatty acid) is known to enhance energy metabolism, immune function, and overall nutrient utilization, which can indirectly support red blood cell synthesis and hemoglobin production [20]. In addition, the decrease in eosinophils suggests a reduced inflammatory response, potentially due to the anti-inflammatory properties of BSFLO or its ability to modulate the immune system. The results are consistent with those of Anas et al. [21], who studied the effect of black soldier fly larvae oil calcium salt (BSFLO-SCa) supplementation on inflammatory response in laying hens and found that BSFLO-SCa treatment enhanced the expression of anti-inflammatory genes and reduced the intestinal pro-inflammation effect associated with immunological function.

Blood biochemical parameters, such as HDL–LDL cholesterol, glucose, and total protein levels, act as indicators for evaluating lipid and protein metabolism in the body. In the current study, the serum HDL cholesterol level was significantly increased by dietary replacement with BSFLO at 75%. This result can be attributed to the high lauric acid content in BSFLO. Shokrollahi et al. [22] reported that using MCFA resulted in enhanced apolipoprotein A1 (ApoA1) secretion, which was correlated with a rise in HDL concentration. ApoA1, the predominant protein component of HDL particles, plays a critical role in modulating HDL concentration. The liver serves as the primary organ responsible for the synthesis and excretion of ApoA1, contributing to 70% of its overall production. This hepatic function is crucial for maintaining adequate levels of ApoA1 in the bloodstream, which, in turn, plays a pivotal role in regulating HDL concentration and cholesterol metabolism [23]. Our results agree with those of German and Dillard [24], who reported in a meta-analysis of 60 trials that dietary lauric acid induced an increase in plasma total cholesterol and HDL.

In addition, decreased AST levels in BSFLO-fed chickens suggest potential benefits for liver health. This could indirectly influence ApoA1 levels by improving liver function. The observed variation in globulin concentration and total protein levels in the bloodstream, particularly at day 28, may indicate altered nutrient metabolism in a short period, potentially a transient effect. However, these effects were observed for a long period at day 63; the BSFLO-fed group exhibited increased serum globulin levels. This increase could be attributed to the immunomodulatory properties of lauric acid, which may stimulate the production of immunoglobulins. Previous reports on the effects of BSFLO on immune function are consistent. Cho et al. [25] reported that after 6 weeks of feeding the BSFL diet to trout, they showed significantly higher expression of immunoglobulin genes compared to fish fed the control diet. Furthermore, the MCFA content of BSFLO might influence lipoprotein metabolism, leading to changes in globulin levels through their incorporation into lipoproteins. MCFA is more ketogenic than LCFA in increasing protein synthesis in the body. Furthermore, MCFA provides an adequate energy supply, thereby diminishing the reliance on protein as an energy source, which increases protein concentration.

Carcass characteristics, such as dressing percentage, edible meat yield, and organ-to-carcass ratios, are essential for assessing poultry production efficiency and meat quality. These measurements provide insights into nutrient utilization, meat yield, and overall health. By analyzing carcass characteristics, producers can optimize their production systems and ensure high-quality meat products. In the current study, the replacement of BSFLO in the diet was 50 and 75%; there was no effect on the carcass or edible meat percentages among all treatments. These findings align with previous studies by Kim et al. [26] and Dabbou et al. [27], which reported that supplementing broiler diets with BSFLO did not affect carcass characteristics.

Meat quality has become a key focus in the poultry industry, as it plays a crucial role in determining the economic and nutritional value of meat products [28]. There are three indicators in determining meat quality, including biological, physical, and chemical qualities [29]. Particularly, physical attributes such as pH, color, cooking loss, and shear force were assessed to evaluate the sensory characteristics of meat quality that influence consumer purchasing decisions. These factors collectively contribute to the overall acceptability and desirability of meat products, ultimately impacting consumer preferences and market demand [30]. Generally, a lower pH typically indicates a higher concentration of lactic acid, which is a byproduct of anaerobic glycolysis, a process that breaks down glycogen to produce energy. In contrast, in the present study, the higher pH at 24 h post-mortem in BSFLO groups suggests lower glycogen storage in muscles at death. Maybe as lauric acid induces the production of ketone bodies, the muscles use this source of energy and store a smaller amount of glycogen in their muscles. Nonaka et al. [31] reported that lauric acid is a medium-chain fatty acid that can be converted into ketone bodies. If muscles are predominantly using ketone bodies for energy, they would rely less on glycogen breakdown, leading to higher pH levels post-mortem.

The study examined the metabolites and pathways influenced by BSFLO supplementation. The metabolomics data indicated that BSFLO affects both nutritional value and muscle metabolism. The BSFLO regulated the metabolite concentrations associated with lipids, amino acids, taurine, and hypotaurine metabolism. Specifically, arginine, a functional amino acid essential for growth, is also the precursor for various bioactive molecules such as nitric oxide, polyamines, agmatine, creatine, glutamine, glutamate, and proline. It also modulates lipid metabolism by reducing total body fat accumulation to improve meat quality and antioxidant defense. Moreover, arginine, a precursor of glutamate, is positively correlated with glutamine, which plays a role in nitrogen balance and promotes protein synthesis [32]. Proline is predominantly synthesized from arginine in animals [33]. Elevated proline levels are associated with a higher nutritional value in meat [34,35,36]. The amino acid content of muscle influences the flavor and nutritional potential of meat [33]. Alanine and glycine can react with reducing sugars to form flavor compounds that enhance meat freshness [37].

Taurine is derived from biosynthesis in methionine and cysteine metabolism, which stabilizes cell membranes and antioxidant properties and is involved in lipid homeostasis [38]. Many studies clarified that taurine plays a significant function in the metabolism of lipids, reduces hepatic and serum lipid accumulation, improves the plasma levels of triglycerides [39,40,41], and enhances both the nutritional composition and quality of meat. De et al. [42] reported that supplementing broiler diets with 0.5% taurine led to a reduction in type IIb muscle fibers in the thigh muscle and also decreased glycolysis, implying a decrease in protein denaturation, which may be contributing to improved meat quality. In the present study, four metabolites associated with L-arginine, L-proline, alanine, glutamine and metabolism were up-regulated. Although this study assessed the impact of these metabolites on meat quality, additional research is necessary to investigate the underlying biological mechanisms.

## 5. Conclusions

This study demonstrates the feasibility of partially replacing RBO with BSFLO in the diets of Thai native chickens without negatively impacting their growth performance. BSFLO supplementation positively influenced blood hematological and biochemical parameters, suggesting potential benefits for overall health and immune function. While BSFLO did not significantly affect carcass characteristics, partially replacing RBO with BSFLO at 50% in the diet could improve meat quality. However, when the substitution level reached 75%, shear force values significantly increased, indicating that higher levels of BSFLO are not conducive to improving meat texture in Thai native chickens. Additionally, BSFLO replacement up-regulated metabolites associated with L-arginine, L-proline, alanine, glutamine, and taurine, suggesting potential improvements in nutritional value and muscle quality. Overall, these findings suggest that, while BSFLO may offer potential benefits for animal health and meat quality in Thai native chickens, its economic viability requires further evaluation. The increased feed costs associated with BSFLO highlight the need for additional research to assess whether these benefits can offset the higher input costs in a commercial enterprise.

## Figures and Tables

**Table 1:** Composition and main characteristic of diets.

Items	Period 1			Period 2			Period 3		
	Control	BSFLO		Control	BSFLO		Control	BSFLO	
		50%	75%		50%	75%		50%	75%
**Ingredient (%)**									
Corn grain	46.10	46.25	45.45	50.50	51.00	51.00	55.75	56.10	56.00
Soybean meal	29.20	29.15	26.95	25.20	21.00	21.00	18.65	16.00	15.20
BSFLO ^a^	0.00	1.05	1.58	0.00	1.60	2.20	0.00	1.95	2.80
RBO ^b^	2.10	1.05	0.53	3.20	1.60	1.00	3.80	1.95	1.00
Full fat soybean	18.00	18.00	21.00	16.60	20.30	20.30	17.80	20.00	21.00
Choline chloride 60%	0.10	0.10	0.10	0.10	0.10	0.10	0.10	0.10	0.10
Limestone	1.60	1.55	1.55	1.50	1.50	1.50	1.40	1.40	1.40
Dicalcium phosphate P21%	1.80	1.75	1.75	1.80	1.80	1.80	1.40	1.40	1.40
Premix ^c^	0.25	0.25	0.25	0.25	0.25	0.25	0.25	0.25	0.25
Salt	0.40	0.40	0.40	0.40	0.40	0.40	0.40	0.40	0.40
DL-Met	0.30	0.30	0.30	0.30	0.30	0.30	0.30	0.30	0.30
L-Lysine	0.15	0.15	0.15	0.15	0.15	0.15	0.15	0.15	0.15
Total	100.00	100.00	100.00	100.00	100.00	100.00	100.00	100.00	100.00
**Calculated nutrient values (%)**									
CP, %	23.00	23.00	23.00	21.00	20.50	20.50	18.94	18.54	18.52
ME, MJ/kg	12.56	12.33	12.29	12.98	12.77	12.62	13.44	13.10	13.00
SFA, %	8.98	9.68	9.95	8.93	11.09	11.49	9.56	11.53	12.37
MUFA, %	14.39	14.72	14.74	14.46	15.80	15.70	15.52	17.11	17.76
PUFA, %	35.13	35.06	34.65	34.42	38.49	38.43	36.64	39.00	40.01

^a^ Black soldier fly larvae oil. ^b^ Rice bran oil. ^c^ Provided per kilogram of diet: 4.80 MIU of vitamin A, 2.00 MIU of vitamin D_3_, 30,000 IU of vitamin E, 1.20 g of vitamin K_3_, 1.20 g of vitamin B_1_, 3.20 g of vitamin B_2_, 2.00 g of vitamin B_6_, 0.0064 g of vitamin B_12_, 24.00 g of niacin, 0.80 g of folic acid, 0.08 g of biotin, and 6.00 g of pantothenic acid; 40.00 g of Zn, 48.00 g of Mn, 16.00 g of Fe, 6.40 g of Cu, 0.50 g of I, 0.04 g of Co, and 0.12 g of Se; 0.20 g of antioxidant, 0.88 g of anticaking, and 1.00 kg of carrier.

**Table 2:** Effects of dietary BSFLO on the growth performance of Thai native chickens.

Productive Performance	Control	BSFLO		*p*-Value	SEM
		50%	75%		
**Days 1–28**					
Initial weight (g/b)	33.44	33.45	33.42	0.45	0.03
Final weight (g/b)	344.89	348.83	346.07	0.93	4.98
Body weight gain (g/b)	311.44	315.38	312.65	0.93	4.98
Feed intake (g/b)	617.50	628.22	658.38	0.54	21.04
Feed conversion ratio	1.98	1.99	2.11	0.59	0.08
Survival rate (%)	100.00	100.00	100.00	NA	NA
**Days 29–42**					
Final weight (g/b)	652.73	658.15	657.17	0.41	8.09
Body weight gain (g/b)	307.84	309.32	311.10	0.31	6.45
Feed intake (g/b)	710.56	730.26	745.78	0.91	51.69
Feed conversion ratio	2.31	2.36	2.39	0.78	0.13
Survival rate (%)	100.00	100.00	100.00	NA	NA
**Days 43–63**					
Final weight (g/b)	1112.68	1115.90	1128.31	0.54	15.53
Body weight gain (g/b)	459.96	457.75	471.14	0.32	9.27
Feed intake (g/b)	1290.85	1282.11	1308.33	0.81	0.65
Feed conversion ratio	2.81	2.80	2.77	0.98	0.12
Survival rate (%)	98.61	98.61	100.00	0.75	1.42
**Days 1–63**					
Body weight gain (g/b)	1079.24	1082.45	1094.89	0.71	19.69
Feed intake (g/b)	2637.43	2657.66	2712.49	0.84	130.93
Feed conversion ratio	2.44	2.46	2.47	0.95	0.09
Survival rate (%)	98.61	98.61	100.00	0.62	1.61
Productive index (%)	69.16	69.04	70.48	0.78	2.26

SEM = standard error of the mean (n = 4); NA = not applicable.

**Table 3:** Effects of dietary of BSFLO on the economic return of Thai native chickens.

Economic Returns	Control	BSFLO		*p*-Value	SEM
		50%	75%		
^1/^SBR (THB/bird)	107.93	108.25	109.49	0.71	1.97
^2/^NPR (THB/bird)	57.06	56.72	58.06	0.76	1.85
^3/^ROI (%)	112.34	110.32	113.56	0.89	6.87
Feed cost (THB/kg)	54.12	54.96	56.39	0.70	2.72
^4/^FCG (THB/kg)	50.86	51.52	51.43	0.93	1.91

SEM = standard error of the mean (n = 4). ^1/^SBR = salable bird return, ^2/^NPR = net profit return, ^3/^ROI return of investment, and ^4/^FCG = feed cost per gain.

**Table 4:** Effects of dietary BSFLO on blood biochemical parameters of Thai native chickens at 28 days of age.

Blood Parameters	Control	BSFLO		*p*-Value	SEM
		50%	75%		
**Blood hematology**					
RBC (×10 ^6^ cells/mm^3^)	2.42	2.52	2.52	0.06	0.04
Hemoglobin (g/dL)	11.50 ^b^	11.78 ^b^	12.80 ^a^	0.001	0.18
Hematocrit (%)	30.00 ^b^	30.33 ^b^	32.25 ^a^	0.001	0.35
WBC (cells/µL)	14,100.00	13,800.00	13,950.00	0.99	1423.97
Heterophils (%)	54.00	48.00	59.33	0.06	3.23
Basophil (%)	0.00	0.00	0.00	NA	NA
Eosinophils (%)	4.33 ^a^	4.00 ^a^	1.00 ^b^	0.03	0.85
Lymphocytes (%)	39.25	34.00	37.00	0.21	2.44
Monocytes (%)	1.00	1.00	1.00	NA	NA
H/L ratio	1.35	1.45	1.52	0.39	0.11
MCV	125.07	121.46	125.52	0.21	1.77
MCH	47.93	48.77	49.74	0.42	1.19
MCHC	38.33 ^b^	39.09 ^ab^	40.00 ^a^	0.05	0.48
**Blood biochemical**					
ALT (U/L)	6.00	5.67	6.00	0.96	1.18
AST (U/L)	235.67	236.33	232.33	0.96	13.75
ALP (U/L)	9505.00	7900.00	6697.00	0.60	2053.90
Glucose (mg/dL)	219.00 ^b^	257.00 ^a^	259.00 ^a^	0.02	9.77
Albumin (g/dL)	1.55	1.55	1.57	0.99	0.11
Globulin (g/dL)	1.73 ^a^	1.67 ^a^	1.47 ^b^	0.04	0.08
Total protein	3.28 ^ab^	3.43 ^a^	3.07 ^b^	0.03	0.09
Triglyceride (mg/dL)	31.00	35.33	33.33	0.69	4.25
Cholesterol (mg/dL)	142.67	147.33	158.33	0.38	9.25
LDL (mg/dL)	30.25	36.33	36.67	0.06	2.37
HDL (mg/dL)	51.33	50.33	50.50	0.99	5.57

SEM = standard error of the mean (n = 12); NA = not applicable. ^a,b^ Means within rows with different superscript letters differ at *p* < 0.05.

**Table 5:** Effects of dietary BSFLO on blood biochemical parameters of Thai native chickens at 63 days of age.

Blood Parameters	Control	BSFLO		*p*-Value	SEM
		50%	75%		
**Blood hematology**					
RBC (×10 ^6^ cells/mm^3^)	2.28	2.59	2.44	0.18	0.13
Hemoglobin (g/dL)	10.48	11.63	10.90	0.36	0.71
Hematocrit (%)	29.50	31.00	30.50	0.54	1.21
WBC (cells/µL)	19,200.00	16,400.00	17,633.00	0.89	4927.99
Heterophils (%)	28.50	27.33	20.00	0.21	3.57
Basophil (%)	2.67	2.25	2.67	0.92	1.14
Eosinophils (%)	1.25	1.00	1.00	0.41	0.21
Lymphocytes (%)	64.75	63.67	70.00	0.40	4.13
Monocytes (%)	1.00	1.00	1.00	NA	NA
H/L ratio	0.45	0.34	0.28	0.92	1.14
MCV	123.91 ^b^	136.37 ^a^	122.40 ^b^	0.03	3.60
MCH	43.87	47.14	44.45	0.13	1.33
MCHC	35.48	34.57	36.40	0.56	1.52
**Blood biochemical**					
ALT (U/L)	4.00	4.00	4.50	0.25	0.23
AST (U/L)	256.33 ^a^	223.67 ^b^	231.50 ^b^	0.01	5.79
ALP (U/L)	4060.00	3687.00	3925.00	0.94	869.89
Glucose (mg/dL)	269.00	268.50	268.25	0.97	4.09
Albumin (g/dL)	1.87	1.83	1.77	0.18	0.04
Globulin (g/dL)	1.93 ^b^	2.25 ^a^	2.25 ^a^	0.01	0.07
Total protein	3.70	3.90	3.93	0.42	0.16
Triglyceride (mg/dL)	34.67	36.33	29.00	0.10	2.55
Cholesterol (mg/dL)	157.00	154.00	168.00	0.41	7.72
LDL (mg/dL)	31.50 ^ab^	30.00 ^b^	32.67 ^a^	0.02	0.56
HDL (mg/dL)	56.00 ^b^	57.33 ^b^	64.33 ^a^	0.05	2.45

SEM = standard error of the mean (n = 12); NA = not applicable. ^a,b^ Means within rows with different superscript letters differ at *p* < 0.05.

**Table 6:** Effects of dietary BSFLO on the carcass quality of Thai native chickens.

Carcass Quality (%)	Control	BSFLO		*p*-Value	SEM
		50%	75%		
**Dressing percentage**	65.45	64.87	64.94	0.08	0.45
**Internal organs** **^1^**					
Wing	15.47	15.40	15.40	0.75	0.25
Thigh	19.42	20.27	19.57	0.07	0.62
Breast	15.86	16.40	16.38	0.21	0.58
Drumstick	16.71	16.79	16.50	0.20	0.25
**Internal organs**					
Liver	2.12 ^a^	1.93 ^b^	2.08 ^a^	0.03	0.12
Heart	0.45 ^b^	0.50 ^a^	0.48 ^a^	0.001	0.02
Pancreas	0.19	0.19	0.20	0.72	0.02
Spleen	0.31	0.34	0.38	0.24	0.07
Abdominal fat	0.44	0.61	0.45	0.06	0.13

^1^ Values expressed as a percentage of carcass weight. SEM = standard error of the mean (n = 16). ^a,b^ Means within rows with different superscript letters differ at *p* < 0.05.

**Table 7:** Effects of dietary BSFLO on the meat quality of Thai native chickens.

Meat Quality	Control	BSFLO		*p*-Value	SEM
		50%	75%		
pH 24 h	5.86 ^b^	5.95 ^a^	5.92 ^a^	0.02	0.02
Color					
L*	55.75	53.71	53.67	0.14	1.65
a*	2.33	1.70	1.57	0.16	0.60
b*	12.04 ^a^	10.27 ^b^	11.65 ^a^	0.03	0.90
Drip loss, %	3.33	3.12	3.89	0.32	0.50
Cooking loss, %	12.47	12.14	13.91	0.65	1.98
Shear force, g/cm^2^	1269.15 ^b^	1151.11 ^b^	1643.49 ^a^	0.0002	103.80
Hardness, g	324.56	307.51	317.51	0.86	38.78

SEM = standard error of the mean (n = 16). ^a,b^ Means within rows with different superscript letters differ at *p* < 0.05.

**Table 8:** Metabolites with significant differences (BSFLO/control group).

Metabolites	Mass-to-Charge Ratio	Formula	Fold Change ^1^	Pathway Analysis ^2^	Regulated
L-arginine	175.12	C_6_H_14_N_4_O_2_	1.24	Arginine biosynthesis	Up
Alanine	90.05	C_3_H_7_NO_2_	1.15	Alanine, aspartate, and glutamate metabolism	Up
Glutamine	147.08	C_5_H_10_N_2_O_3_	1.25	Arginine biosynthesis	Up
L-proline	116.07	C_5_H_9_NO_2_	1.19	Arginine and prolinemetabolism	Up
Taurine	126.02	C_2_H_7_NO_3_S	1.26	Taurine and hypotaurine metabolism	Up

^1^ Fold changes in relative contents between different chickens. Fold change > 1 indicates that the metabolite was down-regulated, whereas fold change < 1 indicates the metabolite was up-regulated. ^2^ The key KEGG pathway of differential metabolites.

## Data Availability

The supporting data for this study are included within the article. For further information or inquiries about the original contributions, please contact the corresponding author.
